# Changes in the number of traffic collisions during the various waves of COVID-19 infection in Japan

**DOI:** 10.1371/journal.pone.0278941

**Published:** 2022-12-15

**Authors:** Ryo Shimada, Kazuhiko Kibayashi

**Affiliations:** Department of Forensic Medicine, School of Medicine, Tokyo Women’s Medical University, Tokyo, Japan; Al Mansour University College-Baghdad-Iraq, IRAQ

## Abstract

An analysis of the national traffic collision trends in Japan for the January 2018 to June 2022 period using existing statistical data indicates that the number of traffic incidents, injuries, and fatalities decreased over time. After the outbreak of COVID-19 in December 2019, traffic volume decreased. In this study, to explore how the COVID-19 pandemic correlates with traffic collisions, we used the Spearman rank correlation of non-parametric statistical test to compare the number of COVID-19 infections with the number of traffic collisions. The number of COVID-19 infections showed a significant inverse correlation with the number of traffic collisions nationwide, in some regions, and in some prefectures. When the number of COVID-19 infections increased, a State of Emergency or Semi-Emergency Spread Prevention Measures were repeatedly declared. We submit that these measures along with the restrictions on the population’s autonomy and movement to prevent the spread of infection, reduces the number of traffic incidents, injuries, and fatalities owing to a decrease in traffic volume. Therefore, these lessons learned from the COVID-19 pandemic advocate that regulation of vehicle traffic volume is an effective means of reducing the occurrence of traffic collisions. These results can be applied to future policy development to support road safety improvements during unique events.

## Introduction

The number of new infections of the coronavirus disease of 2019 (COVID-19) has been increasing worldwide since the Chinese government notified the World Health Organization (WHO) on December 31, 2019 of a case of pneumonia of unknown cause that was detected in Wuhan, Hubei Province. In March 2020, the WHO classified the COVID-19 crisis as a pandemic [1]. As of July 2, 2022, there were approximately 547,500,950 individuals infected with COVID-19 globally and a total number of 6,335,854 deaths had been reported worldwide. As of July 1, 2022 in Japan, there were 9,355,617 individuals who were infected with COVID-19, with 52 of these individuals in a critical condition. An estimated 31,302 deaths have occurred as a result of COVID-19 in Japan and it is estimated that approximately 149,887 individuals have been hospitalized for COVID-19 or are recovering from the illness. Approximately 9,135,363 individuals have been discharged from a medical treatment facility after having COVID-19 [2].

COVID-19 can be transmitted through droplet or contact infection. One individual may infect another when droplets containing the virus are scattered by an infected individual when they cough or sneeze and these droplets are inhaled through the nose or mouth of an uninfected individual. Contact infection occurs when an individual touches an object with infected droplets on it and then touches the mucous membranes of their eyes, nose, or mouth with their droplet-covered hand. It is therefore necessary to pay special attention to public areas where more than one individual touches specific items, such as doorknobs, light switches, and suspension rails, and to try to avoid touching one’s mucous membranes without first washing hands. To prevent the spread of COVID-19, it is recommended that the three “Cs” be avoided. These are: closed or poorly ventilated spaces, crowded places, and close-contact settings where individuals speak to one another in close proximity. Remote working and staggered working hours have also been advised in an effort to curb the spread of the virus [3].

At the end of January 2020, the Japanese government established a COVID-19 task force headed by the prime minister. This task force promoted various measures to limit the spread of the virus and implement programs to deal with the effects of the pandemic. These programs and policies included the designation of COVID-19 as a designated infectious disease and quarantine infectious disease based on the Infectious Diseases Control Law and the Quarantine Act; regulations for immigrants based on the Immigration Control Law; the improvement of medical care provision systems, including returnee and contact counseling centers at public healthcare centers as well as support for businesses and employment sectors [1]. The task force further declared several States of Emergency and implemented Semi-Emergency Spread Prevention Measures to prevent the spread of COVID-19 from April 2020 to March 2022. During these States of Emergency, individuals were asked to refrain from leaving their residence unless absolutely necessary, to cease making use of public facilities, to stop holding events, and to refrain from doing business and trading [1, 4]. During these States of Emergency, movement was restricted and social activities declined, resulting in a decrease in the volume of traffic on the roads [5].

The number of traffic collisions in Japan increased along with the increase in the number of motor vehicles owned, reaching a peak of approximately 718,080 collisions in 1970. Immediately after that, the number of collisions decreased slightly as a result of the oil shock and other factors, but later it began to rise again, reaching a high of 952,720 collisions in 2004. Since then, the number of traffic collisions has decreased steadily, and in 2017, an estimated figure of 472,165 collisions took place. This is approximately half the number of collisions that took place in 2004 [6]. Since the volume of traffic on the roads decreased during the States of Emergency [7, 8], it is possible that the number of traffic collisions that occurred during these periods changed due to increasing COVID-19 infections. A study published in 2021 by one of the authors, Shimada [9], presented data on the number of COVID-19 infections and traffic collisions from January to November 2020 in Japan. The study discussed the relationship between infections and traffic collisions without statistical analysis. From January to November 2020, there were two waves of COVID-19 infections, with the number of traffic collisions decreasing in each wave. However, from December 2020 to June 2022, several more waves of COVID-19 infections occurred. In this study, statistical analysis was performed using the Spearman rank correlation of non-parametric statistical test. We compiled the number of COVID-19 infections and the number of traffic collisions published by the Japanese government and statistically analyzed the relationship between the number of COVID-19 infections and the number of traffic incidents, traffic injuries, and traffic fatalities in Japan from January 2020 to June 2022, for each wave of COVID-19 infection. In addition, data such as population, land area, road area, and number of vehicles owned in each prefecture were also sourced from the Japanese government and used to calculate population density and vehicle density. We statistically analyzed the relationship between population density and vehicle density, and the number of traffic collisions and COVID-19 infections.

We hope that statistical analysis of these interrelationships will lead to a method of regulating traffic collisions. We also expect that the knowledge gained from the results of this study will contribute to the development of policies to support the improvement of traffic safety during future unique events.

## Material and methods

In the previous study by Shimada [9], the number of traffic incidents, traffic injuries, and traffic fatalities that occurred in each month during January 2018 to November 2020, and the number of new COVID-19 infections during January to November 2020 in Japan were extracted. The relationship between the number of COVID-19 infections and the number of traffic collisions from January to November 2020 were presented without statistical analysis. However, in this study, the time period was extended to June 2022 for the number of traffic collisions extracted from January 2018 and the number of COVID-19 infections from January 2020. Furthermore, we calculated the population density and vehicle density for each prefecture. Statistical analysis was performed to determine the correlation between the number of COVID-19 infections, the number of traffic collisions, population density, and vehicle density.

The number of traffic incidents, injuries, and fatalities that occurred in each month for the January 2018 to June 2022 period were extracted from “Table 4–1 Road traffic incidents, fatalities and injuries by prefecture (monthly)” in the “Monthly” portion of statistics relating to the “Traffic Accident Situation” component in the “Statistics about Road Traffic” section published on the E-Stat Portal Site of Official Statistics of Japan [10].

The number of traffic collisions that occur in December of each year is the value for “Table 6–2 Road traffic incidents, fatalities and injuries by prefecture” in the “Yearly” component of the “Traffic accidents situation (December)” part in the “Statistics about Road Traffic” portion [11], minus the value for November of each year as reported in “Table 4–2 Road traffic incidents, fatalities and injuries by prefecture (from the beginning of the year to the present month)” in the “Monthly” portion of the “Traffic Accident Situation” component in the “Statistics about Road Traffic” section published on the E-Stat Portal Site of Official Statistics of Japan [10].

The term “traffic collision” refers to a collision that takes place on a road that is caused by a vehicle, streetcar, or train as is prescribed in Article 2, Paragraph 1, Item 1 of the Road Traffic Act. The term “fatality” refers to the death of an individual that results from a traffic collision and occurs within 24 hours of the collision taking place. The term “injury” refers to both a “serious injury,” which is defined as an injury resulting from a traffic collision requiring medical treatment for 30 days or longer, and a “slight injury,” which requires medical treatment for less than 30 days [12].

The number of new COVID-19 infections for the January 27, 2020 to June 30, 2022 period was extracted from Ministry of Health, Labour and Welfare’s website, especially the page “Visualizing the data: Trend in the number of newly confirmed cases (daily) of information on COVID-19 infections” [13].

The number of traffic collisions, including the number of traffic incidents, injuries, and fatalities that occurred, was extracted for the entire nation, regions, and prefectures for the January 2018 to June 2022 period. In addition, the number of new COVID-19 infections per month for the whole country, regions, and prefectures for the January 2020 to June 2022 period was compared with the number of traffic incidents, injuries, and fatalities that occurred during the same period. The Tokyo prefecture was excluded from the Kanto region and dealt with separately.

The population of the prefectures was extracted from “Table 2–2 Population by Sex, Age (single years) and All nationality or Japanese, and Average age and Median age by Sex and All nationality or Japanese—Japan, Prefectures (DIDs)” of the 2020 population census, published by E-Stat Portal Site of Official Statistics of Japan in October 2020 [14].

The area of the prefectures was based on the “2021 National Survey of area by prefecture, city, town and village (as of October 1)” that was published by the Geospatial Information Authority of Japan, Ministry of Land, Infrastructure, Transport and Tourism on December 21, 2021 [15].

The information in respect of the number of vehicles owned was extracted from the “Table of number of vehicles owned by prefecture and vehicle model (including mini vehicles)” published by the Automobile Inspection & Registration Information Association at the end of September 2021 [16].

The area of the roads in each prefecture was extracted from “Table 4 current status of roads by prefectures (Total)” of road statistics annual report 2020 compiled by the Ministry of Land, Infrastructure, Transport and Tourism [17].

For statistical analysis, the Spearman rank correlation of non-parametric statistical test was used to compare the correlations between the number of COVID-19 infected persons, the number of traffic collisions (including traffic incidents, traffic injuries, and traffic fatalities) that occur, population density, and vehicle density. Statistical analysis was performed using version 16 of the JMP Pro software released by JMP Statistical Discovery LLC in 2021. The differences were considered significant at *p* ≤ 0.05 [18].

## Results

### Overview of national and regional road traffic collisions for the January 2018 to June 2022 period

**Fig 1:**
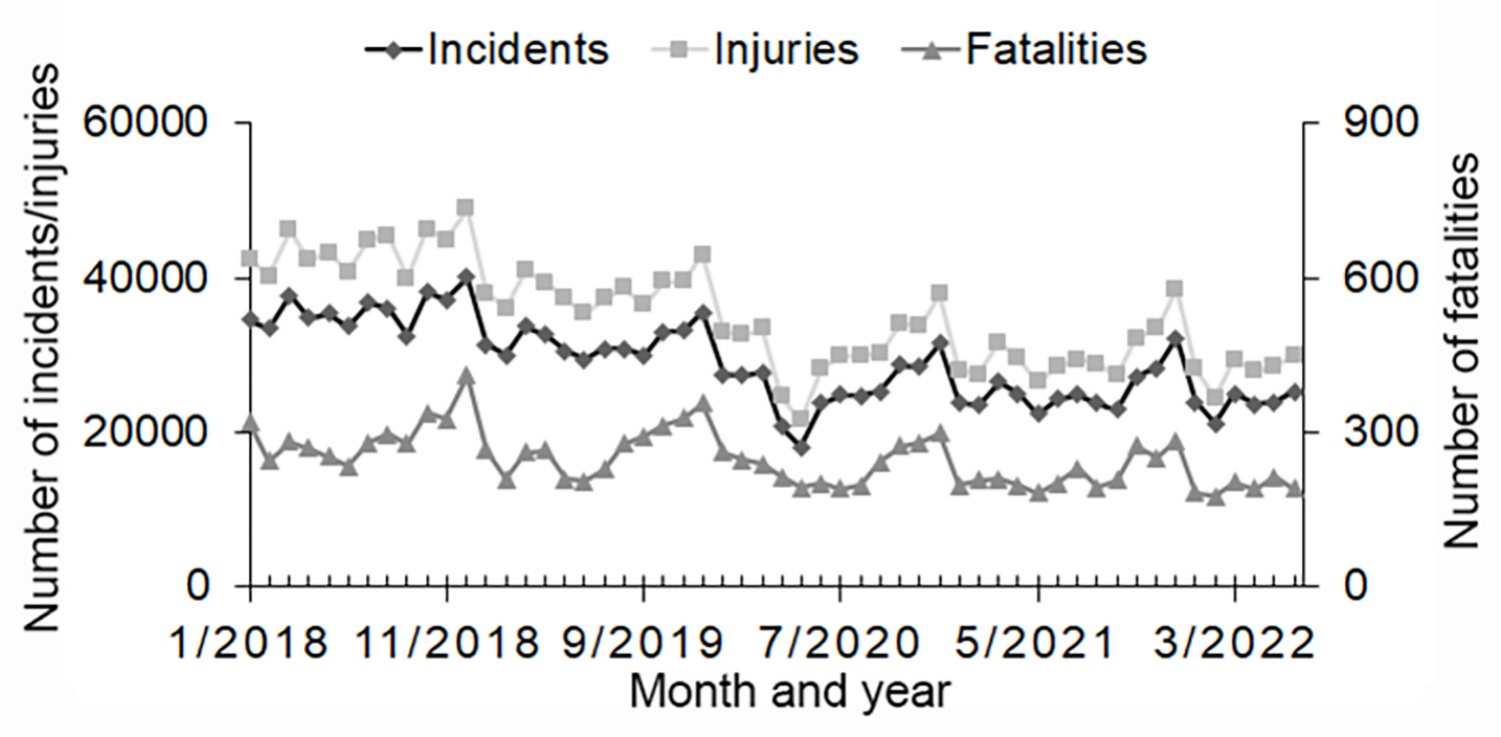
Number of traffic incidents, injuries, and fatalities nationwide during January 2018 to June 2022 period. (Incidents: traffic incidents, injuries: traffic injuries, fatalities: traffic fatalities).

From January 2018 to June 2022, the number of national and regional traffic incidents and injuries decreased year by year and the number of fatalities as a result of national traffic collisions also declined (Fig 1, S1, S2 Figs) However, the number of traffic fatalities in the region varied widely from month to month (S3 Fig).

### National, regional, and prefectural comparisons between the number of COVID-19 infections and road traffic collisions for the January 2020 to June 2022 period

**Fig 2:**
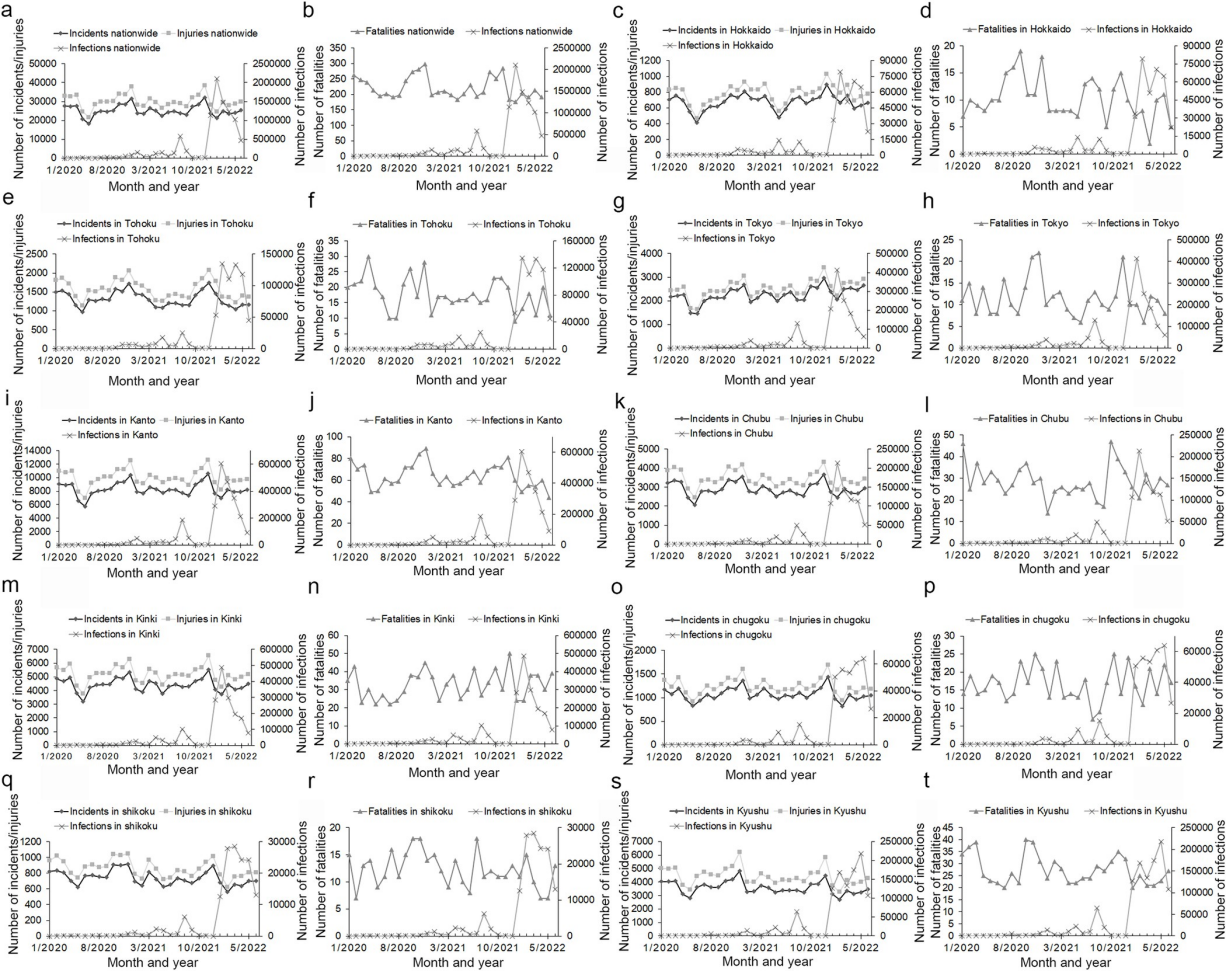
Comparison of number of traffic incidents, injuries, and fatalities with number of COVID-19 infections nationwide, regionally and for Tokyo prefecture for the January 2020 to June 2022 period. (Incidents: traffic incidents, injuries: traffic injuries, fatalities: traffic fatalities, infections: COVID-19 infections).

**Fig 3:**
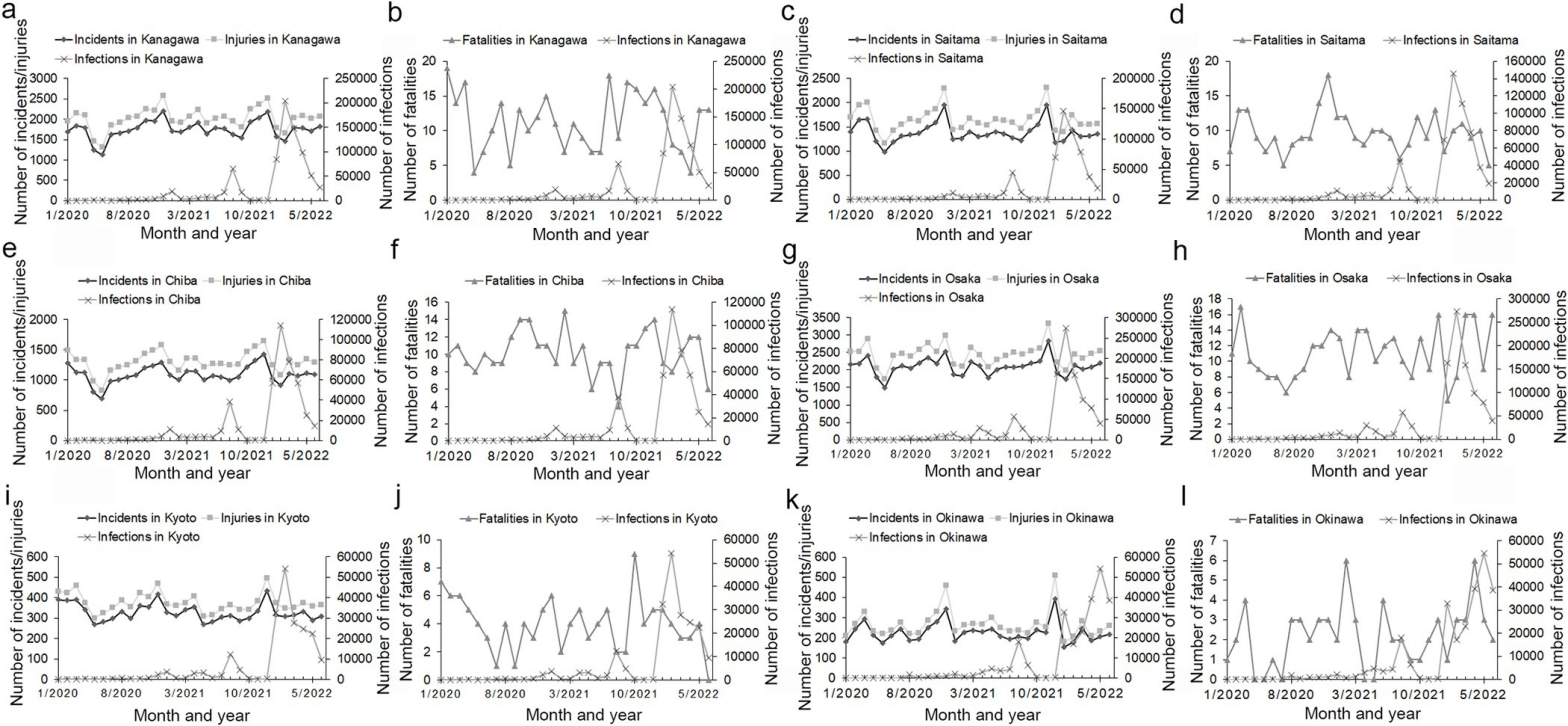
Comparison of number of traffic incidents, injuries, and fatalities with number of COVID-19 infections in high population density prefectures for the January 2020 to June 2022 period. (Incidents: traffic incidents, injuries: traffic injuries, fatalities: traffic fatalities, infections: COVID-19 infections).

**Fig 4:**
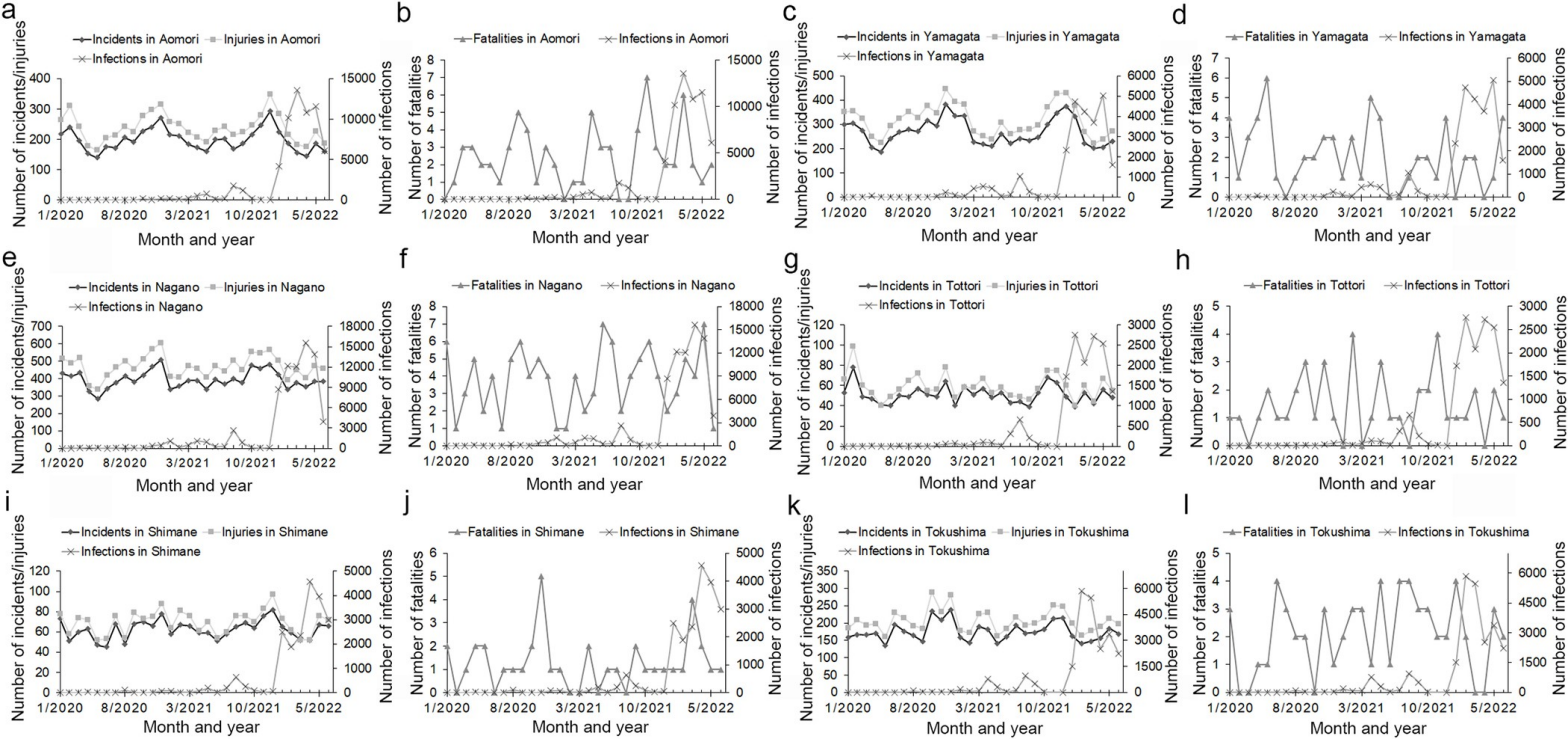
Comparison of number of traffic incidents, injuries, and fatalities with number of COVID-19 infections in low population density prefectures for the January 2020 to June 2022 period. (Incidents: traffic incidents, injuries: traffic injuries, fatalities: traffic fatalities, infections: COVID-19 infections).

During the peak months of COVID-19 infections for the January 2020 to June 2022 period, the numbers of traffic incidents and injuries were found to have decreased to a minimum; further, the number of traffic fatalities showed a downward trend nationwide, as well as in many regions and prefectures, although the number of traffic fatalities varied over the period (Figs 2–4 and S1 Table). The inverse correlation between the number of COVID-19 infections and traffic collisions was significant across the country, as well as in some regions and prefectures (Table 1).

**Table 1:** Correlation coefficient and *p* value for nationwide, region, and prefecture with significant inverse correlation between number of COVID-19 infections and number of incidents, injuries, and fatalities as a result of traffic collisions.

COVID-19 infection	vs. Traffic collision	Spearman rank correlation coefficient (ρ)	*p* value (Prob>|ρ|)
**Nationwide:**			
Infections nationwide	Incidents nationwide	-0.374	0.0418
Infections nationwide	Injuries nationwide	-0.4283	0.0182
Infections nationwide	Fatalities nationwide	-0.5284	0.0027
**Region:**			
Infections in Tohoku	Incidents in Tohoku	-0.4653	0.0096
Infections in Tohoku	Injuries in Tohoku	-0.4893	0.0061
Infections in Tohoku	Fatalities in Tohoku	-0.441	0.0147
Infections in Kanto	Injuries in Kanto	-0.3696	0.0444
Infections in Kanto	Fatalities in Kanto	-0.4382	0.0154
Infections in Chubu	Incidents in Chubu	-0.4287	0.0181
Infections in Chubu	Injuries in Chubu	-0.4434	0.0141
Infections in Chubu	Fatalities in Chubu	-0.5203	0.0032
Infections in Kinki	Incidents in Kinki	-0.3646	0.0476
Infections in Shikoku	Injuries in Shikoku	-0.5758	0.0009
Infections in Kyushu	Incidents in Kyushu	-0.5648	0.0011
Infections in Kyushu	Injuries in Kyushu	-0.5622	0.0012
Infections in Kyushu	Fatalities in Kyushu	-0.472	0.0085
**Prefecture:**			
Infections in Iwate	Incidents in Iwate	-0.4651	0.0096
Infections in Akita	Incidents in Akita	-0.4711	0.0086
Infections in Akita	Injuries in Akita	-0.4378	0.0155
Infections in Akita	Fatalities in Akita	-0.3995	0.0287
Infections in Ibaraki	Incidents in Ibaraki	-0.3665	0.0464
Infections in Ibaraki	Injuries in Ibaraki	-0.376	0.0406
Infections in Niigata	Incidents in Niigata	-0.4897	0.006
Infections in Niigata	Injuries in Niigata	-0.3854	0.0354
Infections in Niigata	Fatalities in Niigata	-0.3984	0.0292
Infections in Yamanashi	Incidents in Yamanashi	-0.464	0.0098
Infections in Yamanashi	Injuries in Yamanashi	-0.4376	0.0156
Infections in Shizuoka	Incidents in Shizuoka	-0.5402	0.0021
Infections in Shizuoka	Injuries in Shizuoka	-0.5136	0.0037
Infections in Shizuoka	Fatalities in Shizuoka	-0.5694	0.001
Infections in Toyama	Incidents in Toyama	-0.3747	0.0414
Infections in Toyama	Injuries in Toyama	-0.4122	0.0236
Infections in Gifu	Incidents in Gifu	-0.3737	0.042
Infections in Gifu	Injuries in Gifu	-0.3982	0.0293
Infections in Aichi	Incidents in Aichi	-0.4132	0.0232
Infections in Aichi	Injuries in Aichi	-0.4532	0.0119
Infections in Mie	Fatalities in Mie	-0.6111	0.0003
Infections in Shiga	Incidents in Shiga	-0.4112	0.024
Infections in Shiga	Injuries in Shiga	-0.403	0.0272
Infections in Hyogo	Incidents in Hyogo	-0.3689	0.0449
Infections in Wakayama	Incidents in Wakayama	-0.6015	0.0004
Infections in Wakayama	Injuries in Wakayama	-0.5303	0.0026
Infections in Hiroshima	Incidents in Hiroshima	-0.4002	0.0284
Infections in Yamaguchi	Incidents in Yamaguchi	-0.5162	0.0035
Infections in Yamaguchi	Injuries in Yamaguchi	-0.5355	0.0023
Infections in Kagawa	Incidents in Kagawa	-0.5963	0.0005
Infections in Kagawa	Injuries in Kagawa	-0.6407	0.0001
Infections in Ehime	Incidents in Ehime	-0.53	0.0026
Infections in Ehime	Injuries in Ehime	-0.5215	0.0031
Infections in Kochi	Injuries in Kochi	-0.637	0.0002
Infections in Fukuoka	Incidents in Fukuoka	-0.5331	0.0024
Infections in Fukuoka	Injuries in Fukuoka	-0.5528	0.0015
Infections in Saga	Incidents in Saga	-0.5742	0.0009
Infections in Saga	Injuries in Saga	-0.5799	0.0008
Infections in Nagasaki	Incidents in Nagasaki	-0.514	0.0037
Infections in Nagasaki	Injuries in Nagasaki	-0.4929	0.0057
Infections in Oita	Fatalities in Oita	-0.3698	0.0443
Infections in Miyazaki	Incidents in Miyazaki	-0.6082	0.0004
Infections in Miyazaki	Injuries in Miyazaki	-0.6117	0.0003
Infections in Kagoshima	Incidents in Kagoshima	-0.6405	0.0001
Infections in Kagoshima	Injuries in Kagoshima	-0.5877	0.0006

Spearman’s rank correlation of non-parametric statistical test analysis was performed using the number of COVID-19 infections, traffic incidents, traffic injuries, and traffic fatalities nationwide as well as in the regions and prefectures. (Infections: COVID-19 infections; incidents: traffic incidents; injuries: traffic injuries; fatalities: traffic fatalities).

Specifically, the first peak of COVID-19 infections occurred nationwide as well as in most regions and prefectures in April 2020. However, the first peak of infections took place in the Tokushima prefecture in March 2020 and the number of COVID-19 infection in the Iwate prefecture was zero from January to June 2020. In the first peak month of COVID-19 infections, the number of traffic incidents, injuries, and fatalities nationwide as well as in the regions, and most prefectures had decreased to a minimum, however, the number of traffic fatalities in the Yamagata, Okayama, Tokushima, and Kochi prefectures increased (Figs 2–4 and S1 Table).

The second peak of COVID-19 infections occurred nationwide as well as in most regions and prefectures in August 2020. However, the second peak occurred in July 2020 in the Aomori, Yamagata, Wakayama, and Kagoshima prefectures, and in July and September 2020 in the Miyagi, Tochigi, and Tottori prefectures. In the second peak month of COVID-19 infections, the number of traffic incidents and injuries were minimized and the number of traffic fatalities decreased nationwide as well as in most regions and prefectures, However, there were increases in some areas: the number of traffic injuries nationwide; the number of traffic incidents and injuries in the Kanto region; the number of traffic incidents in the Kinki region; the number of traffic incidents and fatalities in Hokkaido region; the number of traffic incidents, injuries, and fatalities in Chiba prefecture; the number of traffic incidents and injuries in Kanagawa prefecture; the number of traffic injuries and fatalities in Tottori prefecture; the number of traffic incidents in Gunma, Saitama, and Yamaguchi prefectures; and the number of traffic fatalities in Aichi, Shimane, and Kochi prefectures (Figs 2–4 and S1 Table).

The third peak of COVID-19 infections occurred in January 2021 nationwide, as well as in most regions and prefectures. However, the third peak occurred in November 2020 for the Hokkaido and Tohoku regions, and in December 2020 for the Chugoku region and the prefectures of Iwate, Yamagata, Shimane, Hiroshima, and Kochi. During month of the third peak of COVID-19 infections, the number of traffic incidents, injuries, and fatalities decreased nationwide, as well as in most regions and prefectures (Figs 2–4 and S1 Table).

The fourth peak of COVID-19 infections occurred in May 2021 nationwide as well as in most regions and prefectures. However, the fourth peak occurred in April 2021 in the regions of Kinki and Shikoku and in the prefectures of Yamagata, Nagano, Fukui, Osaka, Hyogo, Nara, Wakayama, Tottori, Tokushima, and Ehime, and in March 2021 in the Miyagi prefecture. In the month of the fourth peak of COVID-19 infections, the number of traffic incidents and injuries decreased and the number of traffic fatalities declined nationwide as well as in most regions and prefectures, except for certain prefectures. The number of traffic injuries in Iwate and Fukui, and the number of traffic fatalities in Aichi increased (Figs 2–4 and S1 Table).

The fifth peak of COVID-19 infections occurred in August 2021 nationwide and for all regions and prefectures. In the month of the fifth peak of COVID-19 infections, the number of traffic incidents and injuries decreased, and the number of traffic fatalities declined nationwide as well as for most regions and prefectures; however, the number of traffic incidents and injuries in Nagano, Shimane, and Kyoto; the number of traffic injuries and fatalities in Okinawa; and the number of traffic fatalities in Yamagata and Tokushima prefectures did not decline (Figs 2–4 and S1 Table).

The sixth peak of COVID-19 infection occurred in February 2022 nationwide and in most regions and prefectures. However, the sixth peak occurred in January and March 2022 in the Shimane, Hiroshima, and Okinawa prefectures. During the sixth peak of COVID-19 infections, the number of traffic incidents and injuries decreased and the number of traffic fatalities declined nationwide, in all regions, and in most prefectures; however, the number of traffic fatalities in the Tottori and Shimane prefectures increased (Figs 2–4 and S1 Table).

### Overview of population density and vehicle density in each prefecture

To verify whether traffic collisions are related to population density or vehicle density, we compiled and compared data on population density and vehicle density for the 47 prefectures (Table 2). The 15 top-ranked and 15 bottom-ranked prefectures in terms of population density and vehicle density were almost identical, except for one top-ranked and two bottom-ranked prefectures, although there were differences in the order. The top 15 prefectures in terms of population density were Tokyo, Osaka, Kanagawa, Saitama, Aichi, Chiba, Fukuoka, Hyogo, Kyoto, Okinawa, Shizuoka, Nara, Hiroshima, Miyagi, and Ibaraki. The top 15 prefectures in terms of vehicle density were almost the same as the top 15 prefectures in terms of population density ranking, although there were also differences in the order. The Ibaraki prefecture, which ranked 15^th^ in population density, was the 28^th^ prefecture in vehicle density. The Shiga prefecture, which ranked 16^th^ in population density, was the 14^th^ prefecture in vehicle density. The bottom 15 prefectures (from 47^th^ to 33^rd^) in terms of population density were Shimane, Iwate, Akita, Kochi, Hokkaido, Yamagata, Nagano, Fukushima, Yamanashi, Tokushima, Tottori, Aomori, Miyazaki, Kagoshima, and Wakayama. The prefectures in the bottom 15 in terms of vehicle density were almost the same as those in the bottom 15 in terms of population density, although there were differences in the order. The Yamanashi prefecture, which was ranked 39^th^ in population density, was ranked 18^th^ in vehicle density and the Wakayama prefecture, which was ranked 33^rd^ in population density, was ranked 20^th^ in vehicle density. The Oita prefecture, which was ranked 30^th^ in population density, was ranked 38^th^ in vehicle density and the Fukui prefecture, which was ranked 31^st^ in population density, was ranked 34^th^ in vehicle density (Table 2). Eleven prefectures in the top ranking and seven prefectures in the bottom ranking failed to show a significant inverse correlation between the number of COVID-19 infections and the number of traffic collisions (Tables 1 and 2; Figs 3 and 4). We found that the area of the prefecture was significantly inversely correlated with population density and vehicle density. The population density and vehicle density in each prefecture were significantly correlated with the total number of COVID-19 infections, traffic incidents, injuries, and fatalities in the three months before and after a peak COVID-19 infection period respectively (Table 3).

**Table 2:** Population density and vehicle density by prefecture in Japan.

Name of prefecture	Population	Land area (km^2^)	Population density (persons/km^2^)	Population density rank	Number of registered vehicles	Road area (km^2^)	Vehicle density (number/ km^2^)	Vehicle density rank
Hokkaido	3973007	83424.41	47.62	43	3809943	434.35	8771.60	44
Aomori	587023	9645.62	60.86	36	1009278	96.03	10510.03	41
Iwate	400246	15275.01	26.20	46	1035510	146.19	7083.32	47
Miyagi	1508978	7282.29	207.21	14	1713602	99.62	17201.39	14
Akita	340699	11637.52	29.28	45	809577	107	7566.14	46
Yamagata	492405	9323.13	52.82	42	934841	80.97	11545.52	36
Fukushima	773118	13784.14	56.09	40	1662395	148.77	11174.26	37
Tokyo	13844009	2194.05	6309.80	1	4427415	126.79	34919.28	3
Ibaraki	1169451	6097.24	191.80	15	2642472	199.48	13246.80	29
Tochigi	929109	6408.09	144.99	20	1748903	119.79	14599.74	20
Gunma	809514	6362.28	127.24	23	1813947	134.64	13472.57	26
Saitama	5998734	3797.75	1579.55	4	4184031	166.87	25073.60	9
Chiba	4823612	5157.31	935.30	6	3707142	157.92	23474.81	10
Kanagawa	8743513	2416.11	3618.84	3	4051706	54.83	73895.79	1
Niigata	1119029	12583.95	88.93	29	1844936	138.06	13363.29	28
Yamanashi	254878	4465.27	57.08	39	769620	48.24	15953.98	18
Nagano	719893	13561.56	53.08	41	1920501	174.31	11017.73	38
Shizuoka	2237324	7777.28	287.67	11	2916720	107.31	27180.32	4
Toyama	414349	4247.54	97.55	28	904273	73.11	12368.66	32
Ishikawa	610464	4186.2	145.83	19	920298	68.42	13450.72	27
Fukui	355428	4190.52	84.82	31	673372	57.79	11652.05	34
Gifu	806499	10621.29	75.93	32	1693477	133.33	12701.40	31
Aichi	5942244	5173.15	1148.67	5	5331269	198.61	26842.90	5
Mie	774064	5774.47	134.05	21	1533092	102.23	14996.50	19
Shiga	754141	4017.38	187.72	16	1055490	61.07	17283.28	13
Kyoto	2176168	4612.2	471.83	9	1345242	50.86	26449.90	7
Osaka	8478518	1905.34	4449.87	2	3819240	69.51	54945.19	2
Hyogo	4306048	8400.94	512.57	8	3059350	137.26	22288.72	11
Nara	887863	3690.94	240.55	12	841069	49.17	17105.33	15
Wakayama	348232	4724.68	73.70	33	761940	52.47	14521.44	21
Tottori	210681	3507.14	60.07	37	470078	39.74	11828.84	33
Shimane	171792	6707.9	25.61	47	557076	72.04	7732.87	45
Okayama	917819	7114.33	129.01	22	1559071	92.58	16840.26	16
Hiroshima	1831138	8479.22	215.96	13	1923254	107.38	17910.73	12
Yamaguchi	683695	6112.55	111.85	27	1075714	77.14	13944.96	23
Tokushima	241941	4146.99	58.34	38	623732	53.95	11561.30	35
Kagawa	314892	1876.92	167.77	17	797643	47.48	16799.56	17
Ehime	720814	5676.12	126.99	24	1032052	75.5	13669.56	24
Kochi	306502	7103.6	43.15	44	567035	54.39	10425.35	42
Fukuoka	3786685	4986.86	759.33	7	3447460	131.34	26248.36	8
Saga	282878	2440.67	115.90	26	689655	51	13522.65	25
Nagasaki	631342	4130.98	152.83	18	963998	74.81	12885.95	30
Kumamoto	865846	7409.39	116.86	25	1406284	98.31	14304.59	22
Oita	547792	6340.7	86.39	30	932397	84.7	11008.23	39
Miyazaki	509617	7735	65.88	35	958559	88.63	10815.29	40
Kagoshima	660703	9186.42	71.92	34	1369238	131.9	10380.88	43
Okinawa	1023230	2282.15	448.36	10	1187318	44.45	26711.32	6

Population density: population/land area (person/km^2^).

Vehicles density: Number of registered vehicles/road area (number/km^2^).

**Table 3:** Correlation between population, land area, road area, number of registered vehicles, population density, vehicle density, number of traffic collisions and number of COVID-19 infection in prefecture.

Peak of COVID-19 infection	Variable	vs. Variable	Spearman rank correlation coefficient (ρ)	*p* value (Prob>|ρ|)
	Land area (km^2^)	Population	0.2810	0.0377
	Population density (persons/km^2^)	Population	0.7439	< .0001
	Population density (persons/km^2^)	Land area (km^2^)	-0.3338	0.0127
	Number of registered vehicles	Population	0.9496	< .0001
	Number of registered vehicles	Land area (km^2^)	0.4472	0.0006
	Number of registered vehicles	Population density (persons/km^2^)	0.5841	< .0001
	Road area (km^2^)	Population	0.6508	< .0001
	Road area (km^2^)	Land area (km^2^)	0.7677	< .0001
	Road area (km^2^)	Population density (persons/km^2^)	0.1263	0.3580
	Road area (km^2^)	Number of registered vehicles	0.8115	< .0001
	Vehicles density (number/ km^2^)	Population	0.6182	< .0001
	Vehicles density (number/ km^2^)	Land area (km^2^)	-0.3911	0.0032
	Vehicles density (number/ km^2^)	Population density (persons/km^2^)	0.9013	< .0001
	Vehicles density (number/ km^2^)	Number of registered vehicles	0.4486	0.0006
	Vehicles density (number/ km^2^)	Road area (km^2^)	-0.0473	0.7315
1	Number of incidents for Mar. to May 2020	Population density (persons/km^2^)	0.6715	< .0001
1	Number of incidents for Mar. to May 2020	Vehicles density (number/ km^2^)	0.5561	< .0001
2	Number of incidents for July to Sept. 2020	Population density (persons/km^2^)	0.6471	< .0001
2	Number of incidents for July to Sept. 2020	Vehicles density (number/ km^2^)	0.5344	< .0001
3	Number of incidents for Dec. 2022 to Feb. 2021	Population density (persons/km^2^)	0.6473	< .0001
3	Number of incidents for Dec. 2022 to Feb. 2021	Vehicles density (number/ km^2^)	0.5372	< .0001
4	Number of incidents for Apr. to June 2021	Population density (persons/km^2^)	0.6767	< .0001
4	Number of incidents for Apr. to June 2021	Vehicles density (number/ km^2^)	0.5640	< .0001
5	Number of incidents for July to Sept. 2021	Population density (persons/km^2^)	0.6636	< .0001
5	Number of incidents for July to Sept. 2021	Vehicles density (number/ km^2^)	0.5529	< .0001
6	Number of incidents for Jan. to Mar. 2022	Population density (persons/km^2^)	0.6691	< .0001
6	Number of incidents for Jan. to Mar. 2022	Vehicles density (number/ km^2^)	0.5630	< .0001
1	Number of injuries for Mar. to May 2020	Population density (persons/km^2^)	0.6657	< .0001
1	Number of injuries for Mar. to May 2020	Vehicles density (number/ km^2^)	0.5494	< .0001
2	Number of injuries for July to Sept. 2020	Population density (persons/km^2^)	0.6552	< .0001
2	Number of injuries for July to Sept. 2020	Vehicles density (number/ km^2^)	0.5436	< .0001
3	Number of injuries for Dec. 2022 to Feb. 2021	Population density (persons/km^2^)	0.6501	< .0001
3	Number of injuries for Dec. 2022 to Feb. 2021	Vehicles density (number/ km^2^)	0.5434	< .0001
4	Number of injuries for Apr. to June 2021	Population density (persons/km^2^)	0.6924	< .0001
4	Number of injuries for Apr. to June 2021	Vehicles density (number/ km^2^)	0.5755	< .0001
5	Number of injuries for July to Sept. 2021	Population density (persons/km^2^)	0.6709	< .0001
6	Number of injuries for Jan. to Mar. 2022	Population density (persons/km^2^)	0.6621	< .0001
6	Number of injuries for Jan. to Mar. 2022	Vehicles density (number/ km^2^)	0.5620	< .0001
1	Number of fatalities for Mar. to May 2020	Population density (persons/km^2^)	0.4792	0.0002
1	Number of fatalities for Mar. to May 2020	Vehicles density (number/ km^2^)	0.3192	0.0175
2	Number of fatalities for July to Sept. 2020	Population density (persons/km^2^)	0.5254	< .0001
2	Number of fatalities for July to Sept. 2020	Vehicles density (number/ km^2^)	0.3912	0.0031
3	Number of fatalities for Dec. 2022 to Feb. 2021	Population density (persons/km^2^)	0.5639	< .0001
3	Number of fatalities for Dec. 2022 to Feb. 2021	Vehicles density (number/ km^2^)	0.4690	0.0003
4	Number of fatalities for Apr. to June 2021	Population density (persons/km^2^)	0.5333	< .0001
4	Number of fatalities for Apr. to June 2021	Vehicles density (number/ km^2^)	0.4181	0.0015
5	Number of fatalities for July to Sept. 2021	Population density (persons/km^2^)	0.5356	< .0001
5	Number of fatalities for July to Sept. 2021	Vehicles density (number/ km^2^)	0.4393	0.0008
6	Number of fatalities for Jan. to Mar. 2022	Population density (persons/km^2^)	0.5934	< .0001
6	Number of fatalities for Jan. to Mar. 2022	Vehicles density (number/ km^2^)	0.4797	0.0002
1	Number of infections for Mar. to May 2020	Population density (persons/km^2^)	0.6443	< .0001
1	Number of infections for Mar. to May 2020	Vehicles density (number/ km^2^)	0.5347	< .0001
2	Number of infections for July to Sept. 2020	Population density (persons/km^2^)	0.7521	< .0001
2	Number of infections for July to Sept. 2020	Vehicles density (number/ km^2^)	0.6195	< .0001
3	Number of infections for Dec. 2022 to Feb. 2021	Population density (persons/km^2^)	0.7579	< .0001
3	Number of infections for Dec. 2022 to Feb. 2021	Vehicles density (number/ km^2^)	0.6342	< .0001
4	Number of infections for Apr. to June 2021	Population density (persons/km^2^)	0.7315	< .0001
4	Number of infections for Apr. to June 2021	Vehicles density (number/ km^2^)	0.6255	< .0001
5	Number of infections for July to Sept. 2021	Population density (persons/km^2^)	0.7633	< .0001
5	Number of infections for July to Sept. 2021	Vehicles density (number/ km^2^)	0.6488	< .0001
6	Number of infections for Jan. to Mar. 2022	Population density (persons/km^2^)	0.7784	< .0001
6	Number of infections for Jan. to Mar. 2022	Vehicles density (number/ km^2^)	0.6498	< .0001

Spearman rank correlation of non-parametric statistical test analysis was performed using the total number of traffic incidents, injuries, fatalities, and COVID-19 infections in the three months before and after the peak COVID-19 infection months. (Incidents: traffic incidents; injuries: traffic injuries; fatalities: traffic fatalities; infections: COVID-19 infections).

## Discussion

This nationwide study of the occurrence of traffic collisions for the January 2018 to June 2022 period using existing statistical data indicates that the number of traffic incidents and injuries has decreased over time. This decrease has been attributed to road safety measures, such as improved vehicle safety performance, increased penalties under the Road Traffic Act and increased road safety guidance, without having regard to the effects of the COVID-19 pandemic [6].

After the outbreak of COVID-19, the number of traffic incidents and injuries decreased further and the number of traffic fatalities showed a downward trend in many places during the peak months of COVID-19 infections (April and August 2020, January, May, August 2021, and February 2022) compared to numbers in 2018 and 2019. However, the number of traffic fatalities varied (S1–S5 Figs).

The number of COVID-19 infections showed a significant inverse correlation with the number of traffic incidents, injuries, and fatalities nationwide; in the Tohoku, Chubu, and Kyushu regions, as well as in the Akita, Niigata, and Shizuoka prefectures. In other regions and prefectures, where significant inverse correlations were found, the number of COVID-19 infections showed a significant inverse correlation with either the number of traffic incidents, and/or injuries and/or fatalities. The peak month of COVID-19 infections was different in some regions and prefectures than in others, and the number of traffic collisions increased inversely during the peak month of COVID-19 infections. However, significant inverse correlations were also obtained. The number of COVID-19 infections within misaligned regions or prefectures showed a significant inverse correlation with the number of traffic injuries and fatalities in the Kanto region; the number of traffic incidents in the Kinki region; the number of traffic injuries in the Shikoku region; the number of traffic incidents and injuries in the Fukushima, Ehime, and Yamaguchi prefectures; the number of traffic incidents in the Iwate and Hiroshima prefectures; and the number of traffic injuries in the Kochi prefecture.

At the time of the first peak of COVID-19 infections in April 2020, the Act on Special Measures against New Influenza (“the Act”) was amended to attempt to prevent the spread of COVID-19 and the basic policy response to COVID-19 was determined on the basis of the Act. Based on the amendments made to the Act, the first State of Emergency was declared on April 7, 2020. The initial State of Emergency covered the following seven prefectures: Tokyo, Saitama, Chiba, Kanagawa, Osaka, Hyogo, and Fukuoka. This was later extended to all prefectures on April 16, 2020. During the first State of Emergency, human contact was required to be reduced by at least 70%, with a goal of an 80% reduction, and the nation was requested to refrain from venturing outdoors, using public facilities, holding events, and conducting business [1, 4]. In April 2020, the month of the first peak of COVID-19 infections, the decrease in the number of traffic incidents, traffic injuries, and the decrease trend in the traffic fatalities may have been as a result of individuals complying with the amendments to the Act in response to the first State of Emergency.

The first State of Emergency was lifted in 39 prefectures on May 14, 2020 and in the following prefectures on May 21, 2020: Kyoto, Osaka, and Hyogo. The first State of Emergency was eventually lifted in the Tokyo, Saitama, Chiba, and Kanagawa prefectures of the capital area and in the Hokkaido region on May 25, 2020 after approximately one and a half months [1]. After the first State of Emergency was lifted, various Go To campaigns, such as Go To Travel and Go To Eat were initiated by the Japanese government to assist the economy to recover as many industries had been adversely affected by COVID-19 pandemic [19]. The second peak in the number of COVID-19 infections occurred in August 2020 and due to fears of a rapid spread of the infection during the New Year’s celebrations, the Japanese government was forced to postpone a number of events [1]. As a result of these measures, the number of traffic incidents and injuries decreased in many prefectures and the number of traffic fatalities showed a downward trend.

In December 2020, the number of new COVID-19 infections in the capital area reached a high level and on January 8, 2021, the Japanese government declared a second State of Emergency for the capital city area, including the Tokyo, Saitama, Chiba, and Kanagawa prefectures, that was terminated on March 21, 2021. Subsequently, a second State of Emergency was declared in the Tochigi, Aichi, Gifu, Kyoto, Osaka, and Hyogo prefectures on January 14, 2021, and this was terminated on February 28, 2021 in all but the Tochigi prefecture (in which it was terminated on February 7, 2021). Unlike with the first State of Emergency, the government did not prevent such a wide range of socio-economic activities in the second State of Emergency, but rather requested restaurants to shorten their trading hours, focusing on situations with a high risk of infection, specifically those involving eating and drinking [1, 20]. Thus, individuals became more aware of the need for restraint, which led to a decrease in the number of traffic collisions during the third peak of COVID-19 infections in January 2021.

By March 2021, the second State of Emergency had been lifted nationwide and the number of COVID-19 infections had stopped decreasing in each prefecture and especially in urban areas, such as Osaka, which exhibited a tendency to rebound. On April 5, 2021 the first Semi-Emergency Spread Prevention Measures were applied to the Miyagi, Osaka, and Hyogo prefectures, but these were gradually extended nationwide as follows: on April 12, 2021 to the Tokyo, Kyoto, and Okinawa prefectures; on April 20, 2021 to the Saitama, Chiba, Kanagawa, and Aichi prefectures; on April 25, 2021 to the Ehime prefecture; on May 9, 2021 to the Hokkaido region and the Gifu and Mie prefectures; and on May 16, 2021 to the Ishikawa, Gunma, and Kumamoto prefectures. As the situation worsened due to the spread of mutated strains of COVID-19 and the healthcare system experienced increased pressure, the government declared a third State of Emergency in the Tokyo, Kyoto, Osaka, and Hyogo prefectures from April 25 to June 20, 2021, and subsequently in the Aichi and Fukuoka prefectures from May 12 to June 20, 2021. The third State of Emergency was declared in the Hokkaido region and the Okayama and Hiroshima prefectures from May 16 to June 20, 2021 as well as in the Okinawa prefecture on May 23, 2021 [20, 21]. The number of COVID-19 infections reached its fourth peak in May 2021, yet the number of traffic collisions nationwide, in the regions, and in most of the prefectures also decreased during the fourth peak month of COVID-19 infections.

The third State of Emergency was lifted nationwide on June 20, 2021, except in the Okinawa prefecture, but the Semi-Emergency Spread Prevention Measures were maintained from June 21 to July 11, 2021 in the Hokkaido region as well as the Tokyo, Aichi, Kyoto, Hyogo, and Fukuoka prefectures and were maintained until August 1, 2021 in the Osaka prefecture. Semi-Emergency Spread Prevention Measures remained in place from April 20 to August 1, 2021 in the Saitama, Chiba, and Kanagawa prefectures without the declaration of a third State of Emergency [20, 21].

As the number of COVID-19 infections increased in Japan, a State of Emergency was declared again. The third State of Emergency in the Okinawa prefecture was only terminated on September 30, 2021, while the fourth State of Emergency was declared in the Tokyo prefecture from July 12 to September 30, 2021 and in the Saitama, Chiba, Kanagawa, and Osaka prefectures from August 2 to September 30, 2021. Semi-Emergency Spread Prevention Measures were implemented in areas where the COVID-19 infections had not yet reached severe levels: in the Hyogo, Kyoto, Fukuoka, and Ishikawa prefectures and in the Hokkaido region from August 2, 2021; in the Gunma, Ibaraki, Tochigi, Shizuoka, Aichi, Shiga, Fukushima, and Kumamoto prefectures from August 8, 2021; in the Miyagi, Mie, Gifu, Hiroshima, Okayama, Toyama, Yamanashi, Ehime, Kagoshima and Kagawa prefectures from August 20, 2021; and in the Kochi, Saga, Nagasaki and Miyazaki prefectures from August 27, 2021. When the number of COVID-19 infections became significant, the fourth State of Emergency was declared: from August 20 to September 30, 2021 in the Kyoto, Fukuoka, Gunma, Ibaraki, Tochigi, Shizuoka and Hyogo prefectures; from August 27 to September 12, 2021 in the Okayama and Miyagi prefectures; from August 27 to September 30, 2021 in the Mie, Gifu, Shiga, Hiroshima and Aichi prefectures and the Hokkaido region. On September 30, 2021, both of the fourth State of Emergency and the Semi-Emergency Spread Prevention Measures were terminated nationwide [20, 21]. However, the number of COVID-19 infections reached its fifth peak in August 2021 and the number of traffic collisions nationwide, in the regions and in most prefectures decreased again in the fifth peak month of the COVID-19 infections.

The sixth peak of COVID-19 infections occurred around February 2022 and the number of COVID-19 infections rose to an unprecedented level. Therefore, Semi-Emergency Spread Prevention Measures were implemented by the government again in the most heavily infected prefectures, beginning on January 9, 2022 and extending to almost all areas nationwide. The government terminated these Semi-Emergency Spread Prevention Measures on March 21, 2022 [21]. Meanwhile, the number of traffic incidents, injuries, and fatalities decreased considerably during the same period. Although there was a downward trend in the occurrence of traffic collisions in January and February each year, there was an even greater decline compared to 2018 and 2019, before the COVID-19 outbreak (S1–S5 Figs).

In the regions and prefectures where there was no inverse correlation between the number of COVID-19 infections and the number of traffic collisions, the number of traffic collisions increased during the peak months of COVID-19 infections. Considering the number of traffic collisions that occurred in 2018 and 2019 before the COVID-19 pandemic, we submit that the number of traffic collisions would not be reduced to nil even if the number of traffic collisions that occurred decreased in the peak months of COVID-19 infections as many prefectures showed a tendency towards an increase in collisions in the months with highest numbers of infections (S1–S5 Figs).

In addition, during the peak months of COVID-19 infections, the number of traffic collisions did not decrease to a minimal amount in many prefectures: the Tokyo, Kanagawa, Saitama, and Chiba prefectures in Kanto region, Osaka prefecture in Kinki regions, Kyoto and Okinawa prefectures, which were among the top 15 prefectures in terms of population density and vehicle density (Fig 3); the Aomori, Yamagata, Nagano, Tottori, Shimane prefectures and the Hokkaido region, which were among the bottom 15 prefectures in terms of population density and vehicle density (Fig 4). We found that if the population density and vehicle density in a prefecture was high, then the number of COVID-19 infections and number of traffic collisions would also be high. It is likely that the number of traffic collisions did not decrease to a minimum because the number of people and vehicles remained high in an area, even if the number of people and vehicles decreased during the peak month of COVID-19 infections in areas with high population and vehicle densities. According to a study conducted in Finland, where both population density and the level of social activity were high, the risk of traffic collisions was also high [22]. In contrast, during the peak months of COVID-19 infections in areas with both low population and vehicle densities, the number of vehicles on the road may have decreased further, making it easier for drivers to achieve higher speeds, which may have led to more traffic collisions occurring. A recent study conducted in Alabama, USA, reported that during the COVID-19 pandemic, the decreased traffic volume on the roads lead to an increase in traffic fatalities as a result of an increase in speeding [23]. It has also been reported that the number of injuries and fatalities due to traffic collisions that occur in sparsely populated areas is inversely related to population density [24].

Since January 2020, the Japanese government has declared multiple States of Emergency and implemented various Semi-Emergency Spread Prevention Measures in order to prevent the transmission of COVID-19. Citizens were urged not to leave their homes unnecessarily and the nation became more aware of the importance of preventing the spread of the virus and reducing activities involving human contact. This resulted in a decrease in the traffic volume compared to previous years [7, 8] and led to a decrease in the number of traffic incidents, injuries, and fatalities. Lessons learned from the COVID-19 pandemic must be applied to future traffic safety measures. In terms of measures to reduce traffic collisions following a pandemic, government-led nationwide actions to reduce traffic collisions, such as encouraging people to walk instead of driving, promoting health by walking, recommending the use of public transportation, and systematically restricting vehicular traffic in areas where traffic crashes occur frequently, will reduce vehicle traffic volume, thereby reducing the number of traffic collisions.

The number of traffic collisions was examined in this study, but not the age structure of casualties; the distinction between drivers, passengers, and pedestrians among casualties; the type of vehicle involved in the collision; or the road on which the collision occurred. A more detailed analysis of these data would result in more targeted countermeasures to traffic collisions. Furthermore, analyzing traffic collisions during the pandemic could aid other countries in developing traffic safety strategies.

## Conclusions

This study used existing statistical data, performed statistical analyses, and compared the results of the analyses to understand the changes in the number of traffic collisions during the various waves of COVID-19 infection in Japan. It was found that from January 2018 to June 2022, the number of traffic collisions that occurred each year exhibited a continuous downward trend. During the peak months of COVID-19 infections, the number of traffic collisions decreased further and reached a minimum. The number of COVID-19 infections showed a significant inverse correlation with the number of traffic incidents, injuries, and fatalities nationwide as well as in some regions and prefectures. Whenever the number of COVID-19 infections increased, a State of Emergency was declared and Semi-Emergency Spread Prevention Measures were issued repeatedly. These measures affected the autonomy of the population and resulted in a decrease in the number of traffic collisions owing to a decrease in traffic volume. Our analysis indicates that controlling the traffic volume is related to a reduction in traffic collisions. Therefore, we submit that the regulation of vehicle traffic volume is an effective measure that can be used to control the occurrence of traffic collisions and subsequent injuries. These lessons learned from the COVID-19 pandemic can be applied to future policy development related to road safety improvements during unique events.

## Supporting information

S1 Fig Number of injuries caused by traffic collisions by region for the January 2018 to June 2022 period. (TIF)

S2 Fig Number of traffic incidents by region for the January 2018 to June 2022 period. (TIF)

S3 Fig Number of fatalities from traffic collisions by region for the January 2018 to June 2022 period. (TIF)

S4 Fig Number of injuries from traffic collisions in prefectures with high population density from January 2018 to June 2022. (TIF)

S5 Fig Number of injuries as a result of traffic collisions in prefectures with low population density for the January 2018 to June 2022 period. (TIF)

S1 Table Peak months for number of people infected with COVID-19 and months of the least number of incidents, injuries, and fatalities of traffic collisions nationwide, region, and prefecture. Infection: Number of people infected with COVID-19. Incident: Number of traffic collision incidents. Injury: Number of injuries due to traffic collisions. Fatality: number of fatalities in traffic collisions. No: The number is zero in the month. The data in the table represents the peak months for the number of COVID-19 infections and the months of the least number of traffic collision, for example “4” means April, “8” means August, etc., “,” means “or”. The name of the region or prefecture appears in the left column of the table.
(XLSX)
