# Supplementary figures and images for: Changes in the number of traffic collisions during the various waves of COVID-19 infection in Japan
Source: PLoS One. 2022 Dec 15;17(12):e0278941. doi: 10.1371/journal.pone.0278941 (PMC9754189; doi:10.1371/journal.pone.0278941)

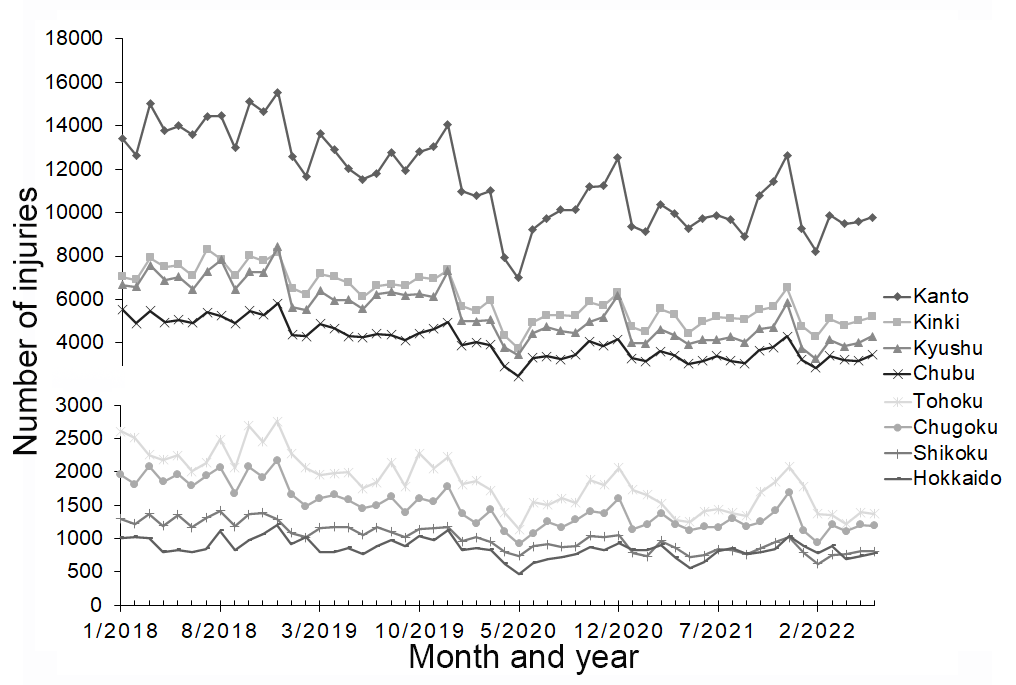

Supplement: S1 Fig — (TIF) [file pone.0278941.s001.tif]

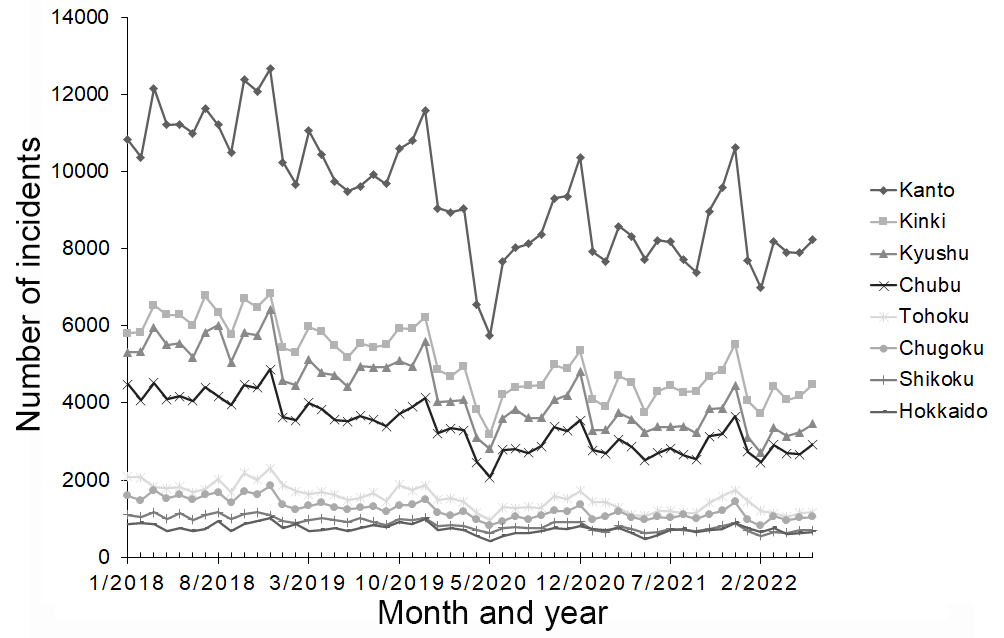

Supplement: S2 Fig — (TIF) [file pone.0278941.s002.tif]

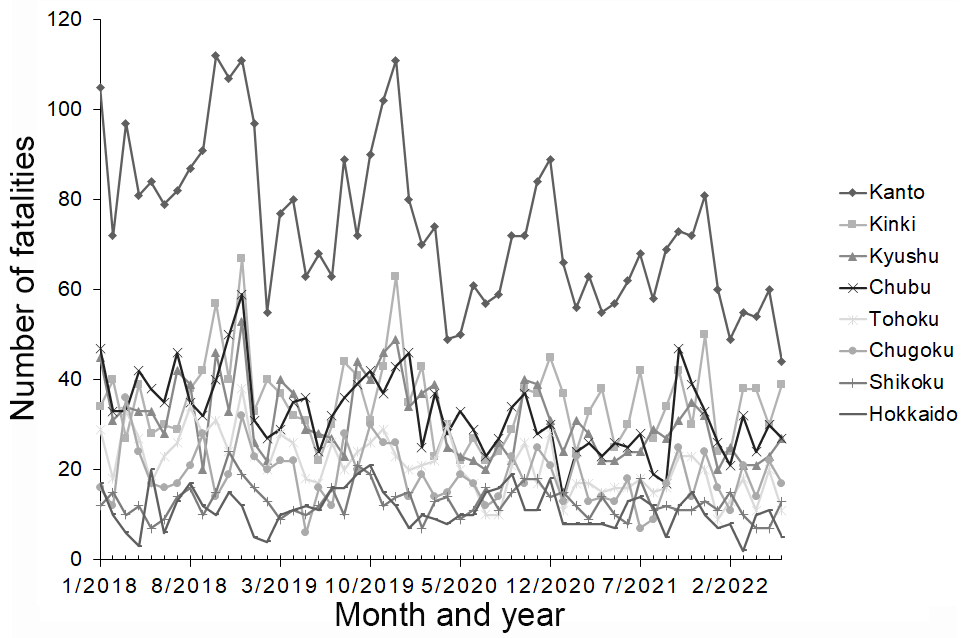

Supplement: S3 Fig — (TIF) [file pone.0278941.s003.tif]

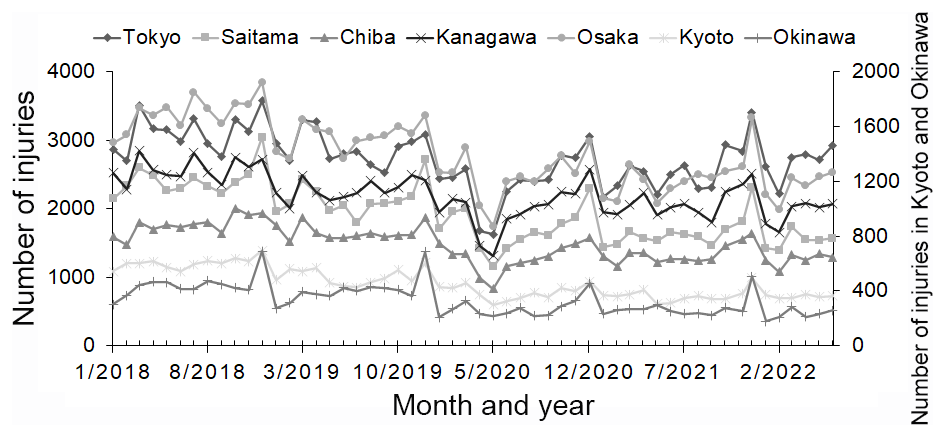

Supplement: S4 Fig — (TIF) [file pone.0278941.s004.tif]

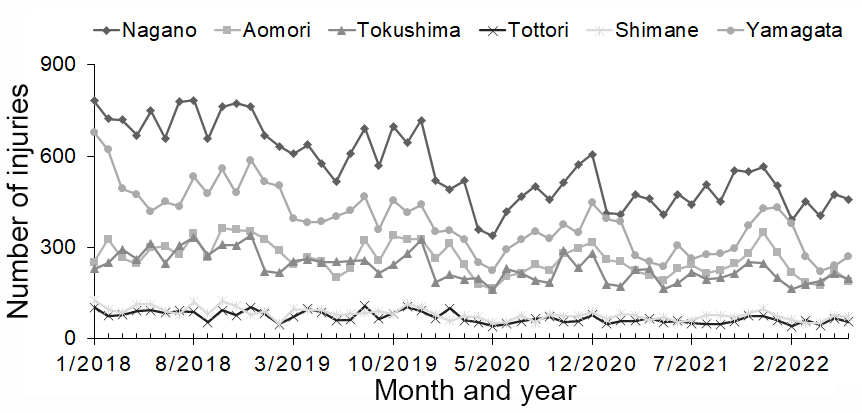

Supplement: S5 Fig — (TIF) [file pone.0278941.s005.tif]
